# Patients with mild cognitive impairment diagnosed at dementia clinic display decreased maximum occlusal force: a cross-sectional study

**DOI:** 10.1186/s12903-021-02027-8

**Published:** 2021-12-27

**Authors:** Hiroyuki Suzuki, Junichi Furuya, Rena Hidaka, Saki Miyajima, Chiaki Matsubara, Gaku Ohwada, Takashi Asada, Chihiro Akazawa, Yuji Sato, Haruka Tohara, Shunsuke Minakuchi

**Affiliations:** 1 Gerodontology and Oral Rehabilitation, Department of Gerontology and Gerodontology, Graduate School of Medical and Dental Sciences, Tokyo Medical and Dental University, 1-5-45 Yushima, Bunkyo-ku, Tokyo, 113-8549 Japan; 2 Department of Geriatric Dentistry, Showa University School of Dentistry, 2-1-1 Kitasenzoku, Ohta-ku, Tokyo, 145-8515 Japan; 3 Dysphagia Rehabilitation, Department of Gerontology and Gerodontology, Graduate School of Medical and Dental Sciences, Tokyo Medical and Dental University, 1-5-45 Yushima, Bunkyo-ku, Tokyo, 113-8549 Japan; 4 Department of Oral Health Sciences for Community Welfare, Graduate School of Medical and Dental Sciences, Tokyo Medical and Dental University, 1-5-45 Yushima, Bunkyo-ku, Tokyo, 113-8549 Japan; 5 Ohwada Dental Office, 1-2-45 Honcho, Kuki City, Saitama 346-0005 Japan; 6 Memory Clinic Ochanomizu, 1-5-34 Yushima, Bunkyo-ku, Tokyo, 113-0034 Japan; 7 Intractable Disease Research Centre, Juntendo University School of Medicine, 2-1-1 Hongo, Bunkyo-ku, Tokyo, 113-8421 Japan

**Keywords:** Mild cognitive impairment, Oral health, Oral status, Oral function, Denture, Maximum occlusal force

## Abstract

**Background:**

Previous research indicates that patients with mild cognitive impairment (MCI) are more likely to have poor oral health and impairments in oral functions, which may be due to few remaining teeth and impaired tongue and lip motor function. However, the oral health of those patients following comprehensive cognitive assessment by a dementia specialist has not been sufficiently investigated. Therefore, this study aimed to clarify the oral function of patients with MCI and the association between oral health and lower cognitive function.

**Methods:**

This cross-sectional study included 96 participants (men: 35; women: 61; mean age: 73.3 ± 8.5 years) who visited a dementia clinic between December 2017 and January 2020. Participants’ cognitive function was assessed by a dementia specialist using neuropsychological and hematological tests and neuroimaging immediately after enrollment. The participants were divided into the healthy and MCI groups according to comprehensive cognitive assessment. Participants’ age, sex, body mass index, primary disease, education level, drinking habits, smoking habits, living environment, employment status, and exercise habits were evaluated. Moreover, oral outcomes, including the number of existing teeth, number of functional teeth (natural and prosthetic teeth which were occluded with antagonists), denture use, oral dryness, tongue and lip motor function, tongue pressure, occlusal force, masticatory ability, and swallowing ability were recorded. The Mann–Whitney U test, χ^2^, and Fisher’s exact tests were used for between-group comparisons. Furthermore, logistic regression analysis using MCI diagnosis as the target variable was performed.

**Results:**

A comprehensive evaluation of the cognitive function of the study participants by the dementia specialist revealed that 48 participants (mean age: 69.8 ± 8.8 years) were healthy and 48 (mean age: 76.9 ± 6.7 years) had MCI. MCI participants were significantly older (p < 0.001) and had significantly fewer existing teeth (p = 0.031) and lower maximum occlusal force (p = 0.019) than healthy participants. Age (odds ratio: 1.126, p = 0.002) and maximum occlusal force (odds ratio: 0.978, p = 0.048) were significantly associated with lower cognitive function.

**Conclusions:**

Patients with MCI had poorer oral health than healthy individuals. Decreased maximum occlusal force was independently associated with lower cognitive function, even when adjusted for age and sex.

## Background

With the increasing age of the population globally, the incidence of dementia is also increasing. According to the 2019 World Health Organization [1] guidelines for dementia, the global population of patients with dementia is approximately 50 million and is increasing by approximately 10 million each year and is expected to reach 152 million by 2050. Similarly, as the number of dementia patients is increasing rapidly in Japan [2], the management of the oral health of older patients with dementia is becoming an increasingly important issue in dentistry [3]. Older adults with dementia are known to have poor oral health, including poor oral hygiene due to inadequate cleaning of teeth and dentures [4, 5], tooth loss due to caries and periodontal disease [6, 7], decreased masticatory ability, difficulties in using dentures [8], and impaired swallowing function [9]. This poor oral health leads to loss of pleasure from eating and impaired quality of life, which increases the risk of undernutrition [10] and aspiration pneumonia [11] along with reduced diversity of meals. Therefore, specialized dental interventions for maintaining good oral health in older adults with dementia are vital [12]. However, implementing this is difficult in many situations [13], such as cases of advanced dementia, due to poor cooperation with dental treatments. Reportedly, various oral factors, such as decreased oral hygiene, periodontal disease, fewer remaining teeth, no denture use, and reduced masticatory ability, are related to cognitive decline [14–17]. As such, oral health should be adequately managed before the onset of dementia.

Mild cognitive impairment (MCI) reflects a state in which a person has normal functioning in activities of daily living and general cognition despite the presence of memory impairment that cannot be explained by age and level of education alone [18]. While 14.4%–55.6% of patients with MCI regain normal cognitive function, the incidence of MCI increases with age, and MCI transitions to dementia in 14.9% of patients diagnosed after the age of 65 [19]. Therefore, in recent years, MCI has been recognized as a stage preceding dementia. At this stage, in which the patient has intact functioning in activities of daily living and general cognition, active oral management including prosthetic treatment can be performed to maintain oral health. In addition, oral health guidance helps maintain and improve cognitive function in older adults with MCI [20]. Therefore, oral health management is critical to maintaining both cognitive and oral functions. Previous research indicates that patients with MCI are more likely to have poor oral health and impairments in oral functions, such as oral diadochokinesis (ODK), which may be due to few remaining teeth and impaired tongue and lip motor function [21–23]. Furthermore, Mizutani et al. [24] conducted a 2-year longitudinal study of older adults without dementia and reported that an increased risk of MCI, as assessed by MCI biomarkers, was associated with decreased masticatory ability. These findings suggest that oral health management by dental professionals is important for patients with MCI. However, MCI was diagnosed in previous studies based solely on the results of a single neuropsychological test, such as the mini-mental state examination (MMSE) [25] or the Japanese version of the Montreal Cognitive Assessment (MoCA-J) [26]. Ideally, diagnosis should be made by a dementia specialist through a comprehensive evaluation of a patient’s cognitive function using hematological [27] and neuropsychological tests, alongside neuroimaging [28, 29]. However, the oral health of the patients diagnosed with MCI in this manner has not been elucidated yet.

Therefore, this cross-sectional study aimed to examine the oral function, in particular oral dryness, tongue and lip motor function, tongue pressure, occlusal force, masticatory ability, and swallowing ability, which are parts of the diagnostic criteria for oral hypofunction [30] of patients diagnosed with MCI by a dementia specialist and to determine the association between oral health and lower cognitive function.

## Methods

### Participants

This cross-sectional study enrolled 111 patients and their family members who visited a dementia clinic with a chief complaint of cognitive decline between December 2017 and January 2020. Family members of the patients were included as participants to minimize the bias of the living environment among the participants as much as possible. The exclusion criteria were as follows: patients who were living in long-term care facilities, patients aged < 50 years, patients who had been diagnosed with dementia, and patients who were unable to undergo an oral function test. Therefore, two patients younger than 50 years, 12 patients diagnosed with dementia, and one patient who was unable to undergo oral function tests were excluded from this study. Finally, the present cross-sectional study included 96 participants. Participants were diagnosed as healthy or having MCI by a dementia specialist through a comprehensive evaluation using neuropsychological tests such as the MMSE [25]; neuroimaging such as electroencephalography, magnetic resonance imaging, cerebral perfusion scintigraphy, and single-photon emission computed tomography; and hematological tests. The general criteria for MCI diagnosis as reported by Winblad et al. were as follows: a person who was judged as neither normal nor demented based on the diagnostic criteria for dementia such as those in the International Classification of Diseases 10 (ICD-10); a person whose functional activities were mainly preserved or minimally impaired; a person with evidence of cognitive decline measured either by self and/or reported by an informant in conjunction with deficits in objective cognitive tasks and/or evidence of decline over time in objective neuropsychological tests [28]. In this study, the diagnostic criteria for MCI were based on these general criteria for MCI. The participants were divided into the healthy and the MCI groups based on a diagnosis of comprehensive cognitive evaluation by a dementia specialist. All participants provided informed consent before enrollment after being given detailed oral and written explanations about the study. This study was approved by the Tokyo Medical and Dental University Faculty of Medicine Ethics Committee (Approval No.: M2017-112).

### Measurements

#### Participants’ basic information

Participants’ age, sex, body mass index (BMI), primary disease, education level, drinking habits, smoking habits, living environment, employment status, and exercise habits were evaluated. Education level, which was classified into the following categories: four-year university graduate, junior college graduate, specialist school graduate, high school graduate, middle school graduate, or other, was used to evaluate each participant’s years of education. This was calculated according to the years of study each category required. Living environment was categorized as either living alone or living with family. Drinking habits, smoking habits, employment status, and exercise habits were categorized as either “Yes” or “No.”

#### Oral health assessment

Oral function was assessed by five dentists and dental hygienists sufficiently trained and proficient in the measurement techniques. Through oral examination, the number of existing teeth, number of functional teeth, and status of denture usage were evaluated. The number of existing teeth was assessed by counting the number of natural teeth, whereas the number of functional teeth which were occluded with antagonists, included prosthetic teeth, such as dentures and implants. Residual roots were excluded from both counts. The number of existing and functional teeth ranged from 0 to 28, respectively. In terms of the status of denture usage, each participant was classified as either using or not using dentures. Additionally, the following oral functions were evaluated. Denture users underwent all tests while wearing dentures.

#### Oral dryness

Oral dryness was evaluated by measuring the wetness at the center of the tongue base using an oral moisture-checking device (Mucus^®^, LIFE Corp., Saitama, Japan) and calculating the median value of three measurements [31]. Oral dryness was defined as a value of less than 27.0 [30].

#### Tongue and lip motor function

ODK while making /pa/ and /ta/ sounds was assessed to evaluate tongue and lip motor function. Participants were instructed to pronounce the syllables /pa/ and /ta/ as many times as possible in 5 s, and the number of syllables per second was measured using an automated measuring device (KENKOUKUN Handy, Takei Scientific Instruments Co. Ltd., Niigata, Japan) [32]. Measurements were carried out twice for each syllable, and the maximum value was regarded as the tongue and lip motor function. Reduced tongue and lip motor function were defined as the pronunciation of /pa/ and /ta/ syllables at a rate of fewer than six times per second [30].

#### Tongue pressure

Tongue pressure was measured using a tongue pressure measurement device (TPM-01, JMS, Hiroshima, Japan). The pressure generated when the balloon of the tongue pressure probe was pressed between the tongue and the palate for approximately 5 s was regarded as tongue pressure [33]. This measurement was performed twice with a 30-s break between measurements, and the maximum value measured was considered the maximum tongue pressure. Low tongue pressure was defined as a maximum tongue pressure of less than 30 kPa [30].

#### Occlusal force

Occlusal force was evaluated using a pressure-sensitive sheet (Dental Prescale, Fuji Film Co., Tokyo, Japan) and an analyzer (Occluzer FPD709, Fuji Film Co., Tokyo, Japan), by measuring the maximum occlusal force at the intercuspal position during clenching for 3 s. The pressure-sensitive sheet was inserted into the participant’s oral cavity, and the participant was instructed to clench down with maximum force at the intercuspal position for 3 s. The maximum occlusal force was calculated by using the analyzer to remove readings (noise) from the region of the pressure-sensitive sheet that was not in the region that the analyzer indicated deviated from the dentition [34]. Reduced occlusal force was defined as a maximum occlusal force of less than 200 N [30].

#### Masticatory ability

Masticatory ability was evaluated using masticatory ability evaluation gum (Xylitol Mastic Gum, Lotte, Tokyo, Japan) that changes color from green to red the more it is chewed [35]. Participants were instructed to chew the gum 60 times at a rate of once per second, and their masticatory ability was evaluated in terms of change in gum color using the visual scoring method with a 10-stage color scale [36]. Reduced masticatory ability was defined as a mastication score of less than six on the color scale [17].

#### Swallowing ability

Swallowing ability was evaluated using the modified water swallowing test (MWST) [37]. Participants were instructed to swallow 3 mL of cold water and their swallowing ability was classified into one of five categories according to the success and condition of swallowing: (1) unable to swallow with choking and/or breathing changes, (2) able to swallow, but with breathing changes, (3) able to swallow with normal breathing, but with choking and/or wet hoarseness, (4) able to swallow with normal breathing and without choking, and (5) in addition to that mentioned for category 4, able to perform twice repetitive swallowing within 30 s. Reduced swallowing ability was defined as an MWST score of < 3.

### Statistical analysis

The Mann–Whitney U test was used to compare the healthy and MCI groups in terms of continuous variables, which included age, BMI, years of education, MMSE, number of existing teeth, number of functional teeth, oral dryness, ODK /pa/, ODK /ta/, maximum tongue pressure, maximum occlusal force, masticatory ability, and swallowing ability. Categorical variables, such as sex and the proportion of participants in each group with reduced oral function were compared using χ^2^ and Fisher’s exact tests. Furthermore, to determine oral function related to lower cognitive function when adjusted for age and sex, a logistic regression analysis was performed using MCI diagnosis as the target variable (healthy = 0, MCI = 1) and age, sex, oral dryness, ODK /pa/, ODK /ta/, maximum tongue pressure, maximum occlusal force, masticatory ability, and swallowing ability as independent variables. Statistical analyses were performed using SPSS Ver. 27 (IBM Japan, Tokyo, Japan), with a significance level of 5%.

## Results

### Participants’ characteristics

A comprehensive evaluation of the cognitive function of the study participants (men: 35; women: 61; mean age: 73.3 ± 8.5 years) by the dementia specialist revealed that 48 participants (mean age: 69.8 ± 8.8 years) were healthy and 48 participants (mean age: 76.9 ± 6.7 years) had MCI. Table 1 represents the basic information of the participants in the healthy and MCI groups. Participants in the MCI group were significantly older (p < 0.001) and had significantly lower MMSE (p < 0.001) than the participants in the healthy group. Although there were significant between-group differences in employment status (p = 0.038) and exercise habits (p = 0.030), there were no significant differences between the groups in terms of the other basic information.

**Table 1:** Participants’ basic information

	Healthy				MCI				p value
	Mean ± SD	Median	n	%	Mean ± SD	Median	n	%	
Age	69.8 ± 8.8	71.5	48		76.9 ± 6.7	78	48		< 0.001*
Sex									
Male			14	29.2			21	43.8	0.138
Female			34	70.8			27	56.2	
BMI (kg/m^2^)	22.5 ± 10.7	22.1	48		22.7 ± 4.0	22.4	48		0.921
Primary disease									
Hypertension			16	33.3			24	50.0	0.098
Cerebrovascular disease			2	4.2			4	8.3	0.399
Heart disease			3	6.3			6	12.5	0.294
Diabetes mellitus			5	10.4			5	10.4	1.000
Hyperlipidemia			10	20.8			9	18.8	0.798
Malignant tumor			3	6.3			7	14.6	0.181
Depression			2	4.2			1	2.1	0.557
Neurologic disease			0	0.0			3	6.3	0.078
Others			17	35.4			24	50.0	0.149
Years of education	14.3 ± 1.8	14	48		13.8 ± 2.5	14	48		0.600
Alcohol									
Yes			22	45.8			22	45.8	1.00
No			26	54.2			26	54.2	
Smoking									
Yes			2	4.2			4	8.3	0.677
No			46	95.8			44	91.7	
Living environment									
Alone			3	6.3			9	18.8	0.064
With family			45	93.7			38	79.2	
Employment									
Yes			25	52.1			15	31.3	0.038*
No			23	47.9			33	68.7	
Exercise habits									
Yes			37	77.1			27	56.2	0.030*
No			11	22.9			21	43.8	
MMSE score	28.7 ± 1.2	29	47		23.8 ± 3.0	24	48		< 0.001**

MCI: mild cognitive impairment, SD: standard deviation, BMI: body mass index, MMSE: mini-mental state examination

*p < 0.05 Healthy group vs MCI group, Chi-square test

**p < 0.05 Healthy group vs MCI group, Mann–Whitney U test

### Oral function

Table 2 represents the oral function results of participants in the healthy and MCI groups. Although the MCI group had significantly fewer existing teeth (p = 0.031) than the healthy group, the fact that denture use was significantly more common in the MCI group (p = 0.025) indicated there was no significant difference between the two groups in terms of the number of functional teeth. Furthermore, the maximum occlusal force was significantly lower in the MCI group (p = 0.019). When assessed based on the diagnostic criteria of oral hypofunction [29], the median values of the parameters of oral function were higher for both groups; however, the MCI group had a significantly higher proportion of participants with impaired ODK /pa/ than the healthy group (p = 0.036).

**Table 2:** Oral status of the participants in the healthy and MCI groups

	Healthy				MCI				p value
	Mean ± SD	Median	n	%	Mean ± SD	Median	n	%	
Number of existing teeth	24.9 ± 5.6	27	48		20.2 ± 9.8	24.5	48		0.031*
Number of functional teeth	27.5 ± 1.8	28	48		27.4 ± 2.0	28	48		0.865
Denture use									
Yes			9	18.8			19	39.6	0.025**
No			39	81.2			29	60.4	
Oral dryness	29.7 ± 3.9	30.6	48		29.4 ± 3.0	30.1	48		0.371
Maintained			43	89.6			39	81.2	0.247
Reduced			5	10.4			9	18.8	
ODK/pa/	6.6 ± 0.6	6.8	48		6.5 ± 0.9	6.6	48		0.586
Maintained			43	89.6			35	72.9	0.036**
Reduced			5	10.4			13	27.1	
ODK/ta/	6.7 ± 0.7	6.6	48		6.4 ± 0.8	6.2	48		0.086
Maintained			42	87.5			35	72.9	0.073
Reduced			6	12.5			13	27.1	
Tongue pressure	33.3 ± 8.1	32.3	48		33.3 ± 7.4	33.3	48		0.878
Maintained			33	68.7			35	72.9	0.653
Reduced			15	31.3			13	27.1	
Maximum occlusal force (/10 N)	41.1 ± 23.4	38.1	48		30.2 ± 21.0	27.9	48		0.019*
Maintained			38	79.2			31	64.6	0.112
Reduced			10	20.8			17	35.4	
Masticatory ability	6.3 ± 1.7	7	48		6.0 ± 1.7	6	48		0.305
Maintained			36	75.0			30	62.5	0.186
Reduced			12	25.0			18	37.5	
Swallowing ability	5.0 ± 0.3	5	48		4.9 ± 0.3	5	48		0.320
Maintained			47	97.9			47	97.9	1.000
Reduced			1	2.1			1	2.1	

MCI: mild cognitive impairment, SD: standard deviation, ODK: oral diadochokinesis

*p < 0.05 Healthy group vs MCI group, Mann–Whitney U test

**p < 0.05 Healthy group vs MCI group, Chi-square test

### Association between oral function and lower cognitive function

Table 3 represents the results of logistic regression analysis. Even after adjusting for age (odds ratio: 1.126, 95% confidence interval: 1.045–1.212, p = 0.002) and sex (odds ratio: 0.528, 95% confidence interval: 0.196–1.423, p = 0.207), maximum occlusal force (odds ratio: 0.978, 95% confidence interval: 0.956–1.000, p = 0.048) was significantly associated with lower cognitive function.

**Table 3:** Results of logistic regression analysis with MCI diagnosis as the dependent variable (Healthy = 0, MCI = 1)

Independent variables	Odds ratio	95% Confidence interval	p value
Age	1.126	1.045–1.212	0.002*
Sex	0.528	0.196–1.423	0.207
Oral dryness	0.979	0.856–1.121	0.762
ODK/pa/	1.297	0.473–3.554	0.613
ODK/ta/	0.710	0.235–2.149	0.545
Tongue pressure	1.027	0.962–1.096	0.420
Maximum occlusal force	0.978	0.956–1.000	0.048*
Masticatory ability	1.142	0.831–1.568	0.413
Swallowing ability	0.896	0.215–3.746	0.881

Sex: Male = 0, Female = 1. All other independent variables were continuous variables. Maximum occlusal force shown per 10 Newton

MCI: mild cognitive impairment, ODK: oral diadochokinesis

*p < 0.05, Logistic regression analysis

## Discussion

The findings of this study revealed that patients with MCI diagnosed by the dementia specialist were older and tended to participate in social circumstances, such as employment or exercise, less frequently than healthy individuals. Furthermore, despite maintained oral function, patients with MCI tended to display reduced maximum occlusal force. Moreover, from the viewpoint of oral hypofunction [30], a high proportion of patients with MCI presented with reduced tongue and lip motor function. Although oral function decreased by aging [38], a reduction in the maximum occlusal force, even when adjusted for age and sex, was independently associated with lower cognitive function. Therefore, the findings of this study suggest that implementing appropriate dental interventions without overlooking subtle changes in oral function such as occlusal force and working with other specialists, such as dementia specialists, might help prevent cognitive decline and the onset of dementia.

Petersen et al. reported that the incidence of MCI increases with age; it is 6.7% between 60 and 64 years, 8.4% between 65 and 69 years, 10.1% between 70 and 74 years, 14.8% between 75 and 79 years, and 25.2% between 80 and 84 years of age [19]. Previous studies have revealed that reduced social participation, which includes activities, such as working [39] and exercise [40] are related to cognitive decline. The participants in the MCI group in this study were also significantly older than those in the healthy group, and the fact that many of them were not working or exercising revealed a trend similar to that discussed in previous reports [39, 40]. Furthermore, the median MMSE score of MCI patients in this study was 24: lower than the cut-off MMSE score [41] for MCI diagnosis. This may be due to the participants in this study having been diagnosed with MCI based on a comprehensive evaluation using neuropsychological tests, such as the MMSE [24], in addition to neuroimaging and hematological tests and because many of them were patients who sought medical attention at a dementia clinic.

Egashira et al. [42] compared the oral function and environment of participants classified into healthy and MCI groups based on MoCA-J scores. They reported that the participants in the MCI group were significantly older than those in the healthy group and that the MCI group had significantly fewer existing teeth, significantly lower masticatory ability, and significantly lower maximum tongue pressure than the healthy group. Further, the lower number of teeth and tongue pressure had a significant impact on the decline in cognitive function, even after adjusting for age. Similarly, in our study the maximum occlusal force of the participants in the MCI group was significantly reduced compared to the healthy group, and, even when adjusted for age and sex, this decrease in maximum occlusal force was significantly associated with lower cognitive function. In a recent study, oral function was demonstrated to decline with age [38]. Therefore, reduced oral function in the MCI group observed in a previous study [42] and the present study may be due to age-related effects. However, both studies reported that poor oral function was independently associated with lower cognitive function, even when adjusted for age or sex, which suggests that oral function may decline in MCI patients regardless of age.

The oral function of participants with MCI was relatively maintained in the context of the diagnostic criteria for oral hypofunction [30]. These results suggest that, while patients with MCI have reduced oral function compared to healthy individuals, this reduction may be mild. Conversely, the percentage of participants with reduced ODK/pa/ was higher in this study than among normal participants, which might be related to the fact that the participants in this study did not participate in social activities, such as employment. Nevertheless, since previous reports suggest that such a decline in oral function may lead to early detection of MCI [21], that a decrease in tongue pressure and ODK are associated with a decreased MMSE score, and that a decrease in maximum occlusal force is associated with a decrease in the MoCA-J score [43], it is important to provide appropriate dental intervention without overlooking subtle changes in oral function for prevention or early detection of dementia. This is an important responsibility of dental practitioners in a super-aged society. In particular, according to Matsubara et al. [20], older adults with MCI who received personalized dental health guidance displayed significant improvement in cognitive function compared to older adults with MCI who did not receive such guidance. Therefore, initiating dental check-ups before MCI onset and appropriately managing oral function in the event of MCI development may be important for maintaining and improving the oral and cognitive functions of older adults with MCI.

This study has several limitations. The first is the potential selection bias that arises from recruiting participants who voluntarily visited a dementia clinic. Since the participants in this study had a relatively high level of health literacy and many were using dentures for missing teeth, they were also considered to have a relatively high level of interest in oral health. Therefore, our findings may not reflect the reality of oral health in all patients with MCI. Additionally, there are some differences in age and other characteristics between the healthy group and the MCI group in this study. A large-scale investigation of community-dwelling older adults, adjusting for background factors where possible, is necessary to elucidate the oral health of patients with MCI. However, it is difficult to perform comprehensive cognitive function tests conducted by a dementia specialist, as done in this study, in all participants of a large-scale investigation. In fact, most previous large-scale studies [21–23] have diagnosed MCI solely based on neuropsychological test results. Neuropsychological tests allow for the easy screening of cognitive function, and while their reliability and validity have been studied [41], some reports indicate that it is difficult to accurately diagnose MCI based solely on neuropsychological test results [44]. As such, in this study, the oral health of individuals who had undergone comprehensive cognitive testing at a dementia clinic and had been diagnosed with MCI by a dementia specialist were examined. Second, oral hygiene, which was one of the diagnostic criteria for oral hypofunction, was not evaluated because the purpose of this study was to examine the kind of oral function that is related to lower cognitive function. In addition, evaluation of chewing and swallowing abilities was done using a different method rather than using the diagnostic criteria for oral hypofunction in favor of simplicity of examination. If the evaluation methods for oral hygiene, chewing ability, and swallowing ability according to the diagnostic criteria for oral hypofunction had been used in this study, the actual condition of oral hypofunction in MCI patients could have been evaluated more accurately. In addition, since this study was not conducted in a dental clinic, evaluation of denture adaptation or periodontal parameters such as plaque index and BOP were not performed because it was considered difficult to accurately evaluate the results. Third, MCI patients were classified into amnestic MCI and non-amnestic MCI based on their symptoms, but this classification was not used in this study. This classification is known to be useful in predicting what type of dementia may develop in the future [45]. Therefore, if MCI patients had been classified into amnestic MCI and non-amnestic MCI in this study and then assessed for their oral function, the oral function associated with each dementia type might have been elucidated. Fourth, the logistic regression analysis excluded the number of existing teeth and the status of denture use as independent variables, despite previous reports demonstrating that these two factors impact cognitive function [15, 16]. However, these two metrics are also known to correlate with occlusal force and masticatory ability [46]; this study confirms this association by indicating significant correlations between the number of existing teeth and maximum occlusal force (r = 0.524, p < 0.001, Spearman’s rank correlation coefficient analysis), between the number of existing teeth and masticatory ability (r = 0.421, p < 0.001, Spearman’s rank correlation coefficient analysis), between the status of denture use (Yes = 0, No = 1) and maximum occlusal force (r = 0.412, p < 0.001, Spearman’s rank correlation coefficient analysis), and between the status of denture use (Yes = 0, No = 1) and masticatory ability (r = 0.367, p < 0.01, Spearman’s rank correlation coefficient analysis). Therefore, to avoid multicollinearity, the number of existing teeth and status of denture use were excluded from independent variables. Moreover, previous research indicates that exercise habits and employment status are associated with cognitive function [39, 40]. In contrast, the decline in social participation represented by these exercise habits and employment status is also reportedly associated with the decline in oral function [47, 48]. In light of these reports, in this study, exercise habits and employment status were not included as independent variables in the logistic regression analysis. Finally, as this was a cross-sectional study conducted at a single center, it could not elucidate the causal relationship between cognitive and oral health changes. Therefore, longitudinal studies are warranted to elucidate the relationship between oral health and cognitive decline in patients with MCI, and interventional studies are needed to determine the most effective oral health intervention to prevent cognitive decline. Moreover, it is extremely important to clarify the cut-off values of oral function that can be used as a reference for MCI diagnosis, and a large-scale survey of MCI patients based on a comprehensive cognitive function assessment is warranted.

## Conclusions

In conclusion, the oral health of patients with MCI diagnosed by the dementia specialist was slightly poorer than that of healthy individuals. In particular, a decrease in the maximum occlusal force was independently associated with lower cognitive function, even when adjusted for age and sex. The findings of this study suggest that for prevention of cognitive decline, it is important to implement appropriate oral management without overlooking even the most subtle changes in oral health.

## Data Availability

All data generated or analyzed during this study are included in this published article.

## Abbreviations

MCI: Mild cognitive impairment
BMI: Body mass index
ODK: Oral diadochokinesis
MMSE: Mini Mental State Examination
MoCA-J: Japanese version of the Montreal Cognitive Assessment
MWST: Modified water swallowing test
